# Evaluation of the Correlation of Serum Copeptin Levels Patients Diagnosed with Gastrointestinal System Bleeding with the Stage of Hemorrhagic Shock

**DOI:** 10.5152/eurasianjmed.2026.251248

**Published:** 2026-06-06

**Authors:** Ayça Çalbay, Murat Maksut Çalbay, Sultan Tuna Akgöl Gür, Atıf Bayramoğlu, Nurinnisa Öztürk, Bülent Albayrak

**Affiliations:** 1 Department of Emergency Medicine, Atatürk University Training and Research Hospital, Erzurum, Türkiye; 2 Ministry of Health, Erzurum Provincial Health Directorate, Erzurum, Türkiye; 3 Alaaddin Keykubat University, Antalya, Türkiye; 4 Department of Medical Biochemistry, Atatürk University Training and Research Hospital, Erzurum, Türkiye; 5 Department of Internal Medicine, Atatürk University Training and Research Hospital, Erzurum, Türkiye

**Keywords:** Copeptin, gastrointestinal bleeding, hemorrhagic shock, replacement

## Abstract

**Background:**

The relationship between serum copeptin (CPT) levels and the stage of hemorrhagic shock and the need for fluid/blood replacement in patients with gastrointestinal system (GIS) bleeding has not yet been clearly established. Therefore, this study was planned to evaluate the relationship between serum CPT levels and the stage of hemorrhagic shock and the need for fluid/blood replacement in patients diagnosed with GIS bleeding.

**Methods:**

This prospective, single-center, observational study was conducted on a total of 90 patients; 43 of these were patients with GIS bleeding, and 47 were in the control group (peptic ulcer). Serum CPT levels were measured using the enzyme-linked immunosorbent assay method. Stages of hemorrhagic shock were determined according to the Advanced Trauma Life Support 11th edition. Hemoglobin, hematocrit, mean**corpuscular ** volume, international normalized ratio, lactate, base deficit, and replacement needs were determined and recorded.

**Results:**

While there was a statistically significant difference between serum CPT levels and the blood parameters studied in patients with GIS bleeding, only the stages of hemorrhagic shock did not statistically affect CPT levels. Increased fluid and blood product replacement was observed in advanced shock stages.

**Conclusion:**

Elevated serum CPT levels in patients with GIS bleeding may be a prognostic biomarker reflecting the severity of the disease. However, it is not sufficient on its own for determining hemorrhagic shock stages and predicting replacement needs.

**Main Points**

- Serum copeptin (CPT) and basal deficit values were significantly higher in patients with gastrointestinal system (GIS) bleeding, while hemoglobin (Hb) levels were significantly lower.
- Serum CPT levels, which increase in correlation with the increasing hemorrhagic stage in patients diagnosed with GIS bleeding, gain prognostic importance.
- Correlation analyses conducted to determine the relationship between serum CPT levels and the laboratory parameters studied revealed a negative correlation between CPT and Hb, and statistically significant positive and weak correlations between CPT and mean corpuscular volume, lactate, and international normalized ratio values.
- The CPT levels can be used in the follow-up of GIS bleeding and in prognosis prediction.

## Introduction

Gastrointestinal system (GIS) bleeding is a common medical situation resulting in high patient morbidity and healthcare costs.^1^ Gastrointestinal system bleeding, which can range from vague to severe bleeding in terms of clinical symptoms, can affect the entire GIS, including the pancreas, liver, and bile ducts.^2^ The clinical presentation of acute gastrointestinal bleeding can range from a nonspecific, non-pathognomonic state resembling fatigue to massive bleeding characterized by hemorrhagic shock.^2^ Bleeding is divided into 2 groups according to its source: upper and lower GIS bleeding. The most common cause of upper gastrointestinal bleeding is peptic ulcers, mostly originating from duodenal ulcers.^3^^,^^4^ Among the pathologies that can cause upper GIS bleeding is esophageal variceal bleeding, which has a high mortality rate and is a complication of portal hypertension.^5^ The most common anatomical cause of lower GIS bleeding is diverticulosis, accounting for 15%-55% of cases.^6^^,^^7^ Among vascular causes, angiodysplasias are prominent in patients over 65 years of age,^8^^,^^9^ while hemorrhoids are more prevalent in patients under 50 years of age.^10^ In the previous study, the incidence of GIS bleeding in adults was approximately 170 cases per 100 000 population, and it was higher in the elderly. It was more common in men than in women.^11^

Arginine vasopressin (AVP), also known as antidiuretic hormone, is a neuropeptide stored in the posterior pituitary gland and has endocrine, hemodynamic, and osmoregulatory effects.^12^^,^^13^ Produced in the hypothalamus and transported to the posterior pituitary by neurophysin 2, arginine vasopressin is released into the circulation following hemodynamic and osmotic stimuli. It exerts its peripheral effects on V1a, V1, and V2 receptors.^12^^,^^13^ Arginine vasopressin plays an important role in maintaining intravascular volume and pressure and is also released in response to stress.^14^^-^^16^ The body responds immediately to sudden, life-threatening situations by releasing AVP and CPT.^17^ Arginine vasopressin is essential for maintaining vasomotor tone during shock and supports vasoconstriction via non-adrenergic pathways.^18^ Particularly in animal studies, it has been shown that during hemorrhagic shock, 10%-20% of AVP is rapidly released from the posterior pituitary gland, where it is stored, and there is a marked acute increase in circulating levels.^19^^,^^20^ The CPT, located at the C-terminal end of AVP, is a long glycated peptide consisting of 39 amino acids with a leucine-rich core.^21^ The CPT is more stable in circulation than AVP and is therefore easier to quantify or preserve.^22^ The CPT concentrations correlate with AVP levels in both healthy volunteers and critically ill patients.^23^^,^^24^ The fact that only a small amount of plasma or serum is required for measurement and that it can be measured within 20-30 minutes facilitates the use of CPT in clinical practice.^25^

The relationship between serum CPT levels and the stage of hemorrhagic shock and the need for fluid/blood replacement in patients with GIS bleeding has not yet been clearly established. Therefore, this study was planned to evaluate the relationship between serum CPT levels and the stage of hemorrhagic shock and the need for fluid/blood replacement in patients diagnosed with GIS bleeding.

## Material and Methods

### Study Limitations

This study is that only patients who presented to the emergency department with GIS bleeding between the study dates were included.

### Ethical Approval and Participants

This study was designed as a single-center, observational, prospective study conducted between September 15, 2023, and June 15, 2024, and was approved by the Atatürk University Faculty of Medicine Scientific Research Ethics Committee with decision number B.30.2.ATA.0.01.00/603 dated September 7, 2023. Informed consent was obtained from the volunteers participating in the study. The study was conducted in accordance with the Helsinki Declaration and good clinical practice guidelines. The study included 120 patients who presented to the emergency department with complaints such as bloody vomiting, black stools, or fresh blood in the stool. Fifteen of these patients were excluded because their clinical management in the emergency department centered on sepsis (n = 15), pulmonary embolism (n = 10), or cardiopulmonary arrest (n = 5). The remaining 90 patients underwent diagnostic endoscopy. Among them, 43 were diagnosed with gastrointestinal bleeding, while 47 were diagnosed with peptic ulcer and were assigned to the control group. The choice of peptic ulcer as the control group was based on the study by Salt et al,^28^ which reported an association between the frequency of peptic ulcer diagnosis and serum CPT levels among the etiological factors of GIS bleeding.

The inclusion criteria for the study were being at least 18 years of age, not being pregnant, and having endoscopically confirmed gastrointestinal bleeding. The control group consisted of patients who presented to the emergency department with complaints of bloody vomiting, black stools, or fresh blood in the stool, but whose clinical, laboratory, and endoscopic findings did not support gastrointestinal bleeding and who were newly diagnosed with peptic ulcer. These patients were over 18 years old and had no diagnosis of acute cerebrovascular disease, myocardial infarction, cardiac or respiratory arrest, decompensated heart failure, pulmonary embolism, aortic dissection, sepsis, or acute respiratory distress syndrome. Shock stages were determined according to the 11th edition of the Advanced Trauma Life Support guidelines. Informed consent was obtained from all patients in both the case and control groups.

### Blood Analysis

For biochemical analysis, blood was allowed to clot for 10 minutes, then centrifuged at 3000 rpm for 10 minutes, and the serum was separated. Serum international normalized ratio (INR) and lactate levels were measured using a Beckman Coulter AU 5800 device. Blood gas (base deficit) was measured using an ABL800 FLEX device. Hemoglobin (Hb), hematocrit (Htc), and mean corpuscular volume (MCV) levels were measured using a Mindray BC-6800 Plus device. The CPT (Cat. No. ABT1470Hu, Atlas Biotechnology Ltd., Ankara, Türkiye) levels in serum were prepared according to the manufacturer’s standard protocol using an enzyme-linked immunosorbent assay (ELISA) kit and measured on an ELISA reader (BioTek PowerWave XS, USA). All samples were tested twice, and the means were used for statistical analysis. The intra-assay and inter-assay variation coefficients of the kit were less than 10%.

### Statistical Analysis

Statistical analyses were performed using SPSS version 20.0 (SPSS, Chicago, IL, USA). The normality of parameters was assessed using the Kolmogorov–Smirnov test. Comparisons for parameters showing normal distribution were performed using analysis of variance and independent samples *t*-test (Student’s *t*-test). For parameters not showing a normal distribution, the Kruskal–Wallis and Mann–Whitney *U-*tests were used. The chi-square test was used for comparing categorical data. Results were expressed as mean ± SD and minimum-maximum values. A *P* value <.05 was considered statistically significant.

## Results

**Figure 1.**
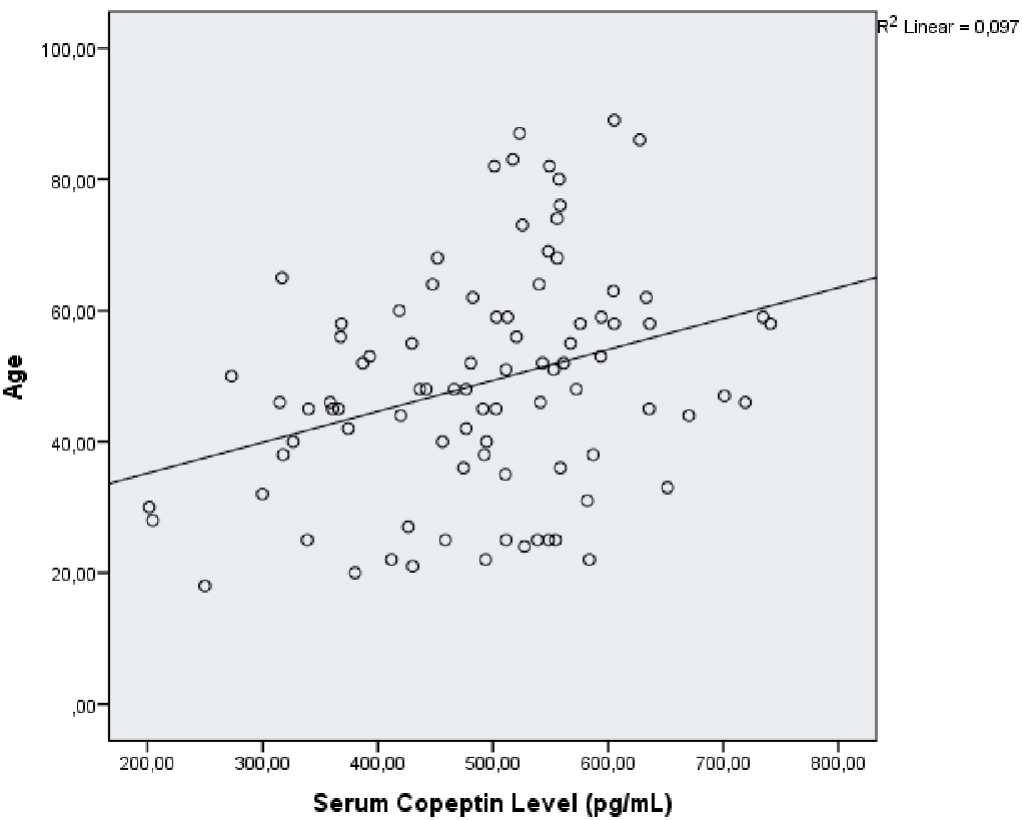
Correlation graph between serum copeptin levels and age.

Serum CPT and basal deficit values were significantly higher (*P* = .001) in patients with GIS bleeding, while Hb levels were significantly lower (*P* = .004). International normalized ratio values were higher in the case group (0.001). When looking at the mean age of the study population, it was determined that the mean age of patients with GIS bleeding was higher than that of the control group (*P* = .001) (Table 1). A statistically significant positive correlation was determined between serum CPT values and age (Figure 1) (*P* = .003, *r* = 0.3129).

**Figure 2.**
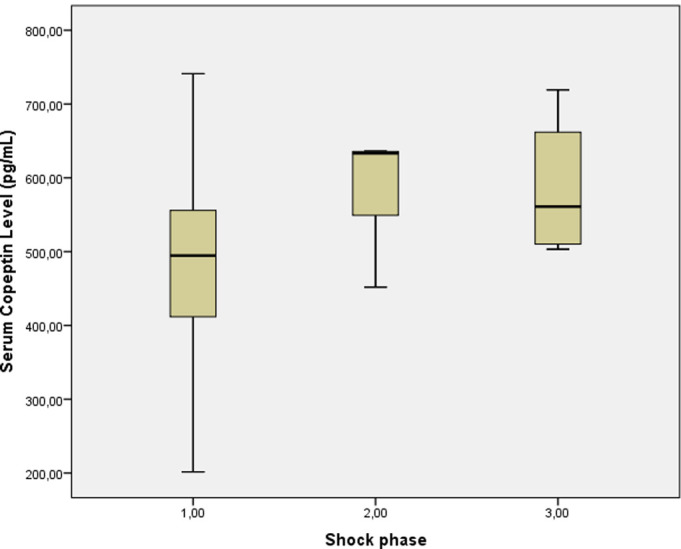
Correlation graph between serum copeptin levels and hemorrhagic shock stages.

**Figure 3.**
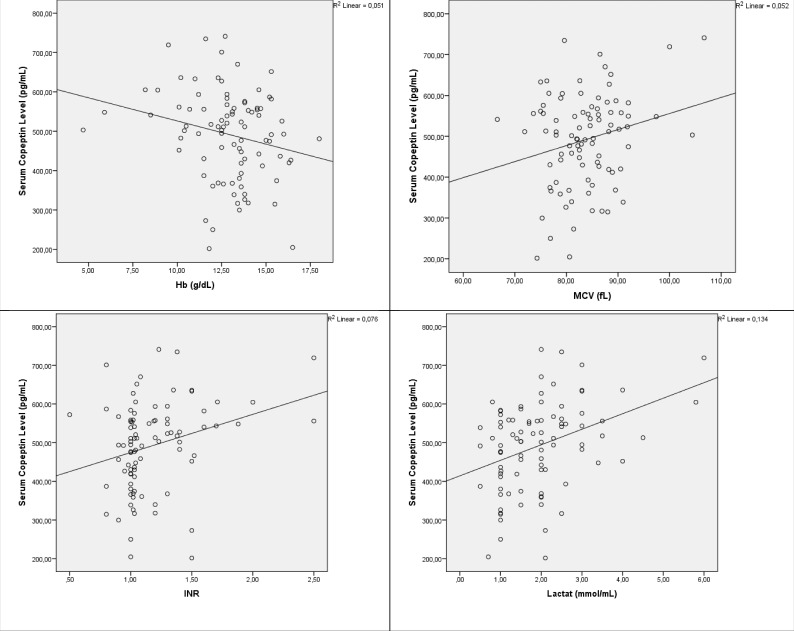
Correlation graphs between serum copeptin levels and routine laboratory parameters.

When patients were grouped according to hemorrhagic shock stage in the study, there was no statistically significant difference in terms of gender between shock stages 1, 2, and 3 (*P* = .329) (Table 2). When comparing the correlation between hemorrhagic shock stages and serum CPT levels, no statistically significant determinant was found. When patients with GIS bleeding were evaluated according to shock stages using laboratory parameters other than CPT, Hb, Htc, MCV, lactate, and INR levels, except for base deficit, were found to be statistically significant and predictive between hemorrhagic shock stages 1 and 3 (*P* = .001). The base deficit value was shown to be predictive in the analysis between shock stages 2 and 3 (*P* = .005) (Figures 2 and 3; Tables 3 and 4).

Correlation analyses conducted to determine the relationship between serum CPT levels and the laboratory parameters studied revealed a negative correlation between CPT and Hb, and statistically significant positive and weak correlations between CPT and MCV, lactate, and INR values (Figure 3, Table 4).

**Figure 4.**
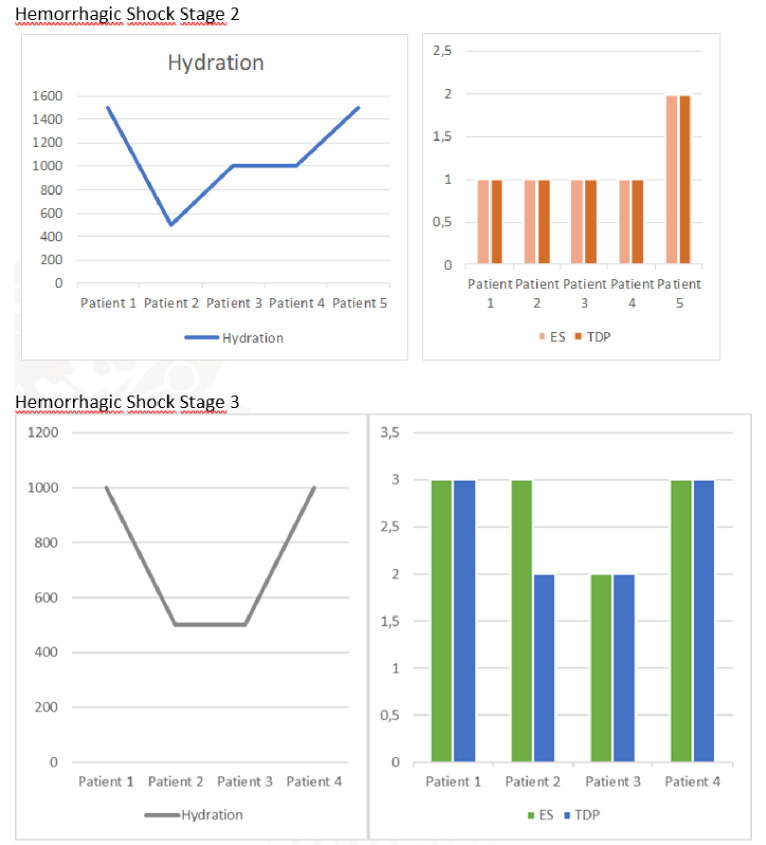
Hemorrhagic shock stage 2 and 3 fluid resuscitation table (ES, erythrocyte suspension-unit; FFP, fresh frozen plasma-unit; hydration-cc).

Fluid and blood resuscitation administered to patients in advanced shock stages is as shown in Figure 4.

## Discussion

Recent studies have shown that CPT is a diagnostic and prognostic factor in many different diseases such as pneumonia, heart failure, hemorrhagic and septic shock, and that its levels increase proportionally with the severity of the disease.^26^^,^^27^ Therefore, the relationship between serum CPT levels and hemorrhagic shock stages in patients with gastrointestinal bleeding was investigated. Although serum CPT levels were higher in patients with gastrointestinal bleeding compared to the control group, it was shown that these levels did not have a statistically significant value as the hemorrhagic shock stages progressed. In the study, patients taking medications and those with comorbidities were excluded because serum CPT levels may be affected. Previous studies have shown that most patients have additional diseases that can alter mortality rates; however, CPT levels have not been found to be useful in determining the prognosis in patients with gastrointestinal bleeding.^28^

While serum CPT levels increased, both shock and biochemical parameters showed a significant decrease in Hb and Htc values. This situation may be related to the fact that blood loss/decreased blood pressure in GIS bleeding increases arterial hypovolemia^29^ or a strong CPT response due to baroreceptor stimulation.^30^ Solà et al^31^ reported that baroreceptor stimulation occurred due to the presence of significant arterial hypovolemia and portal hypertension resulting from splanchnic vasodilation. The findings were consistent with these results. Additionally, the increase in CPT suggests that it could be a monitorable biomarker for tracking the severity of a patient’s clinical condition if GI erosion increases and causes damage that may lead to bleeding. A negative correlation was observed between CPT levels and complete blood count parameters such as Hb, Htc, and MCV, also in the stages of hemorrhagic shock. This is thought to lead to activation of the AVP mechanism and increased CPT release. Struck et al^32^ reported that there was no significant relationship between hemoglobin and blood pressure values and CPT values. It has also been reported that this may be due to the fact that the amount of bleeding is not excessive and that shock has not developed in patients who present to the emergency department. The difference between the findings and the data in the literature is thought to be related to the fact that patients presenting to the emergency department had lost large amounts of blood and were in the shock phase. A strength of this study is that it adds a supportive dimension to the limited number of studies showing a strong correlation between CPT and patients with GIS bleeding.

In the study, a positive correlation was found between INR and serum CPT levels. Patrick et al^33^showed a positive correlation between INR and diseases with poor prognosis and high mortality rates. In other words, they determined that INR values increased with increasing patient mortality or worsening prognosis. Afzal et al^34^reported that high INR values, indicating impaired clotting, are frequently observed in patients with chronic liver disease and are significantly associated with more severe bleeding. Previous studies have shown that the increase in INR values is due to infections affecting the coagulation pathways.^33^^,^^35^ Similarly, a positive correlation was observed between lactate and base deficiency values and CPT levels. Increased bleeding in the patient will lead to a decrease in circulating blood volume and the development of ischemia. A decrease in circulating blood volume will increase oxygen deficit, metabolic acidosis will be observed, and lactic acid in the blood will increase. It is thought that as the patient’s blood loss and oxygen deficit increase, the amount of lactic acid in the circulation will also increase. Bakker et al^36^ reported that in patients with hypovolemic shock, the mortality rate increased from 18% to 73% when blood lactate levels were above 4 mmol/L. This confirms the effect of bleeding severity on mortality.

In the study, the high number of patients in the initial stage of hemorrhagic shock, the early development of significant symptoms during bleeding, and the fact that many patients seek medical attention before the onset of shock, thus reducing the incidence of advanced-stage shock, all contribute to a higher risk of rapid deterioration in the hemodynamic status of patients in the severe hemorrhagic shock stage. Therefore, in patients with advanced-stage shock, the rapid deterioration of tissue perfusion, changes in consciousness, and loss of physical strength can reduce their likelihood of seeking medical help and accessing healthcare systems, potentially leading to a rarity of advanced-stage cases.

In conclusion, this study demonstrated the potential of serum CPT as a prognostic biomarker in patients with GIS bleeding, as its levels showed significant correlations with blood values such as hemoglobin, lactate, MCV, and INR. However, CPT levels were insufficient to show a significant difference between hemorrhagic shock stages. Therefore, while CPT can support the clinical assessment of gastrointestinal bleeding, it is not a reliable marker alone for determining the stages of hemorrhagic shock. Larger studies are needed to further clarify its clinical utility.

## Figures and Tables

**Table 1.** Comparison of Laboratory Parameters Between Case (Gastrointestinal Bleeding) and Control (Peptic Ulcer) Groups

Parameters	Patients with GIS Bleeding (n = 43)	Patients with Peptic Ulcer (n = 47)	*P*
Age (Years)	57.02 ± 17.23	41.55 ± 13.80	.001*
Hb (g/dL)	12.23 ± 2.45	13.55 ± 1.72	.004*
Htc (%)	37.87 ± 6.80	40.99 ± 3.65	.007*
MCV (fL)	84.67 ± 7.78	82.73 ± 5.27	.165
PLT (10^3^/Ul)	186 ± 77.52	204.36 ± 65.36	.226
Lactate	2.38 ± 1.21	1.52 ± 0.64	.001*
INR**	0.50-2.50	0.80-1.60	.001*
Base deficit**	−17-3.0	−3.0-4.0	.001*
CPT (pg/mL)	559.15 ± 74.74	429.93 ± 109.87	.001

*Statistically significant *P* value, **Parameters that do not show a normal distribution, min-max values are given. For normally distributed values, mean ± standard deviation is given.

CPT, copeptin; GIS, gastrointestinal system; Hb, hemoglobin; Hct, hematocrit; INR, international normalized ratio; MCV, mean corpuscular volume; PLT, platelet count.

**Table 2.** Comparison of Gender and Shock Phase of Gastrointestinal System Bleeding

	Shock Phase GIS Bleeding			
Gender	1	2	3	Total
Male	21	1	3	25
Female	15	3	1	19
Total	36	4	4	44

GIS, gastrointestinal system.

**Table 3.** Laboratory Parameters and Statistical Significance Levels According to Hemorrhagic Shock Stages

Parameters	Shock Phase 1 (n = 81)	Shock Phase 2 (n = 5)	Shock Phase 3 (n = 4)	*P*
Age (Years)	47.39 ± 17.03	63.0 ± 13.56	62.75 ± 15.33	.119^a^ .182^b^ .999^c^
Hb (g/dL)	13.22 ± 1.95	11.34 ± 1.32	8.75 ± 1.82	.101^a^ .001**^b^** .129^c^
Htc (%)	40.27 ± 4.92	36.08 ± 2.17	28.35 ± 8.45	.169^a^ .001^b^ 0.060^c^
MCV (fL)	83.25 ± 6.04	82.42 ± 7.08	93.45 ± 11.20	.956^a^ .001^b^ .030^c^
PLT (10^3^/Ul)	202.16 ± 69.93	127.20 ± 55.92	148 ± 74.91	.056^a^ .286^b^ .896^c^
Lactat	1.74 ± 0.78	3.14 ± 0.94	4.20 ± 2.12	.002^a^ .001^b^ .172^c^
INR	0.5-2.5	1.15-1.50	1.23-2.5	.110^a^ .001^b^ .131^c^
BD**	−7.0-4.0	−5.0-3.0	−17.0-−1.0	.013^a^ .001^b^ .005^c^
CPT (pg/mL)	81.48 ± 113.17	581.21 ± 81.27	586.06 ± 99.44	.133^a^ .165^b^ .998^c^

*Parameters that do not show normal distribution, min-max values are given. For normally distributed values, mean ± standard deviation is given.

BD, base deficit; CPT, copeptin; Hb, hemoglobin; Hct, hematocrit; INR, international normalized ratio; MCV, mean corpuscular volume; PLT, platelet count.

^a^Shock Phase 1 vs. Shock Phase 2.

^b^Shock Phase 1 vs. Shock Phase 3.

^c^Shock Phase 2 vs. Shock Phase 3.

**Table 4.** Correlation Analysis Between Copeptin and Hemoglobin, Mean Corpuscular Volume, Lactate, International Normalised Ratio Values

		Hb (g/dL)	MCV (fL)	INR*	Lactate (mmol/mL)
CPT (pg/mL)	*P*	.033	.031	.003	.001
	*r*	−.226	.227	.306	.367

*Spearman Correlation was performed.

CPT, copeptin; Hb, hemoglobin; INR, international normalized ratio; MCV, mean corpuscular volume.

## Data Availability

The data that support the findings of this study are available on request from the corresponding author.
